# DUA: A Domain-Unified Approach for Cross-Dataset 3D Human Pose Estimation

**DOI:** 10.3390/s23177312

**Published:** 2023-08-22

**Authors:** João Renato Ribeiro Manesco, Stefano Berretti, Aparecido Nilceu Marana

**Affiliations:** 1 Faculty of Sciences, UNESP—São Paulo State University, Bauru 17033-360, SP, Brazil; nilceu.marana@unesp.br; 2 Media Integration and Communication Center (MICC), University of Florence, 50134 Florence, Italy; stefano.berretti@unifi.it

**Keywords:** 3D human pose estimation, domain adaptation, adversarial neural networks

## Abstract

Human pose estimation is an important Computer Vision problem, whose goal is to estimate the human body through joints. Currently, methods that employ deep learning techniques excel in the task of 2D human pose estimation. However, the use of 3D poses can bring more accurate and robust results. Since 3D pose labels can only be acquired in restricted scenarios, fully convolutional methods tend to perform poorly on the task. One strategy to solve this problem is to use 2D pose estimators, to estimate 3D poses in two steps using 2D pose inputs. Due to database acquisition constraints, the performance improvement of this strategy can only be observed in controlled environments, therefore domain adaptation techniques can be used to increase the generalization capability of the system by inserting information from synthetic domains. In this work, we propose a novel method called Domain Unified approach, aimed at solving pose misalignment problems on a cross-dataset scenario, through a combination of three modules on top of the pose estimator: pose converter, uncertainty estimator, and domain classifier. Our method led to a 44.1mm (29.24%) error reduction, when training with the SURREAL synthetic dataset and evaluating with Human3.6M over a no-adaption scenario, achieving state-of-the-art performance.

## 1. Introduction

Human pose estimation is an important and challenging computer vision problem. Its objective is to estimate the human body shape (pose) based on a single image, usually monocular. This shape can be inferred by the detection of joints in a skeleton, which are connected in such a way that each connection represents a part of the human body [1].

Two-dimensional (2D) human poses can be employed in a diverse and vast set of applications, of major relevance to society, among which we can mention crowd control, action recognition, person identification, medical aid for therapies and sports analysis, human–computer interaction, augmented and virtual reality, and pedestrian location for autonomous cars [2].

Regarding 3D human poses, there are a few ways to approach the estimation problem: for example, by using depth sensors, infrared sensors, radio sensors, or even multiple camera perspectives by pose triangulation. However, these solutions end up being costly to implement or work in highly controlled environments [2]. Besides those restrictions, with the growth of digital cameras shipped in mobile devices, like smartphones and webcams, a necessity to approach the 3D human pose estimation problem by using monocular RGB images does emerge.

While the labeling process in 2D poses can be simply obtained by the usage of crowd-sourcing tools, like Amazon Mechanical Turk (https://www.mturk.com/, accessed on 24 March 2022), obtaining 3D pose labels requires the usage of motion capture systems in restricted environments [3], decreasing the diversity of the image data present in the training set and aggravating the issue with 3D pose estimation methods based on RGB monocular images.

**Figure 1:**
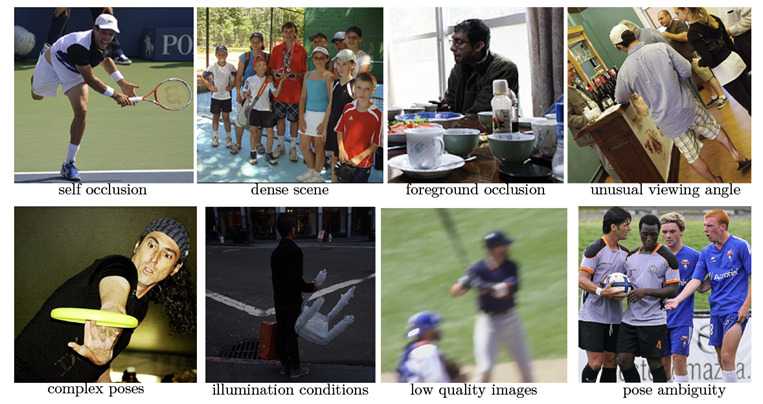
Examples of challenges that can be found in the human pose estimation task in real environments (domains) extracted from the MS-COCO dataset [5].

The usage of a single RGB camera introduces several challenges to the problem of human pose estimation, such as, for example, the occurrence of occlusions and the lack of full-body images of some individuals. Furthermore, variations in clothing, body type, and camera angle can have a negative impact on the performance of the methods [4]. In Figure 1, it is possible to notice some of the challenges in human pose estimation from images captured in real and non-controlled environments (domains). These challenges become more severe when methods based on a single RGB image are used.

A solution to this problem involves taking advantage of the maturity of 2D pose estimation methods to obtain the 3D pose in two steps: a first step, where the 2D pose is obtained using traditional methods, such as OpenPose [6], PifPaf [7] or Stacked Hourglass [8], followed by a second step, where from a 2D pose input the 3D pose is estimated.

According to Martinez et al. [9], state-of-the-art 3D two-step pose estimation techniques tend to perform better than their end-to-end counterparts. Even so, recent methods have reported high levels of overfitting regarding camera angles in frequently used databases, thereby impacting the performance of real applications [10,11,12,13,14]. Furthermore, evaluation protocols aimed to address these issues are rarely used in the literature, such that the most frequently used workaround is data augmentation across different camera angles.

An alternative, and arguably more reliable solution to this problem, would be to include synthetic datasets during the training step. These options enable an increase in the variety of poses, scenes, and camera angles that are available, providing a way to deal with the overfitting problem caused by the use of a single dataset. Doing in this way, however, introduces a shift from the real domain to the synthetic image one [15].

Despite recent demonstrations of improvement in human pose estimation, the lack of approaches in the inter-domain scenario for two-step methods is still perceptible, with solutions unable to address all the nuances of cross-domain 3D human pose estimation. The following issues were identified as exacerbating the domain discrepancy in the task:

- The utilization of diverse body capture sensors across distinct datasets that leads to distinct pose representations, consequently resulting in misalignment between joints;
- Distinct domains frequently exhibit misalignment in their camera and action distributions, which can impact the accuracy and robustness of 3D human pose estimation;
- The propagation of errors on the edge kinematic groups, namely the arms and legs, resulting in a substantial increase in the overall error.

To address the aforementioned problems, and subsequently mitigate the domain discrepancy, this study introduces several improvements to the pose estimation procedure. The following contributions form a crucial part of this work and will be discussed along this paper:

- The introduction of a pose conversion technique aimed at achieving a unified pose representation to overcome the observed differences between capture sensors;
- An enhancement and evaluation of the pose estimation training pipeline through the development of a novel uncertainty-based method;
- The creation of a domain adaptation model based on adversarial networks for 3D human pose estimation.

**Figure 2:**
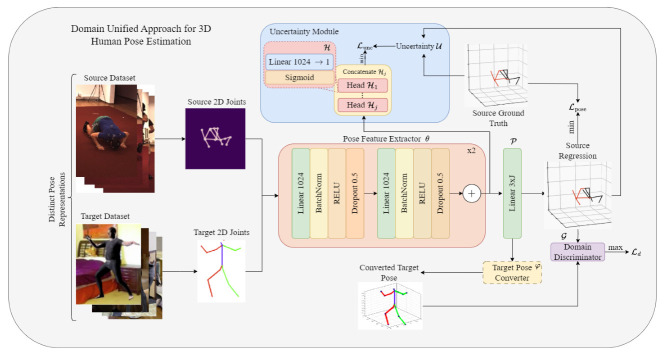
Proposed domain-unified approach for 3D human pose estimation. The method is composed of three main modules on top of the 3D pose estimator: the uncertainty estimation module, the pose conversion module, and the domain discriminator. The dashed lines on the pose converter represent frozen weights.

Figure 2 summarizes our method and its contributions. Our method, called the domain-unified approach (DUA) for 3D human pose estimation, works in a cross-domain scenario, where the goal is to learn from labeled 3D source data and generalize effectively to an unsupervised target domain.

The method is structured around three main modules, all operating on top of a backbone pose estimator. Initially, the pose estimator serves as a feature extractor, from which one can obtain poses from a dedicated pose head P. These extracted features are fused with pose predictions to generate an uncertainty estimate, aiding the training process. Furthermore, the predicted target-domain poses undergo transformation into a unified pose representation, harmonizing joint distribution with the source domain. Lastly, a domain discriminator is employed, tasked with distinguishing between source and target poses. Its role is to facilitate the establishment of a consistent feature representation within a common domain.

## 2. Related Work

According to Stamou et al. [16], a human pose can be described as an articulated body, that is, an object composed of a set of rigid parts connected through joints, which allow the execution of translational and rotational movement in six degrees of freedom. Therefore, human pose estimation is a problem that aims to find a particular pose *P* in a space Π that contains all possible articulated poses. In the context of RGB images, the objective is to extract a set of features from the image that represent each joint of the human body.

Although there are commercial solutions that address the 3D pose estimation problem, these solutions work mostly in restricted environments, as is the case of those based on Kinect, which has a depth sensor, or use markers for body detection [2], which ends up being quite restrictive. Therefore, there is a need to propose more flexible solutions to 3D pose estimation, which can be used in uncontrolled scenarios, preferably using a low-cost easily accessible monocular RGB camera. This is particularly challenging because depth information should be recovered from a single image.

The increasing availability of 2D pose data has provided a viable solution to address the scarcity of annotated 3D poses by employing a two-step human pose estimation approach. Earlier works in two-step 3D human pose estimation aimed at obtaining the poses through Euclidean distance matrices [17], but the potential of the technique was elevated when Martinez et al. [9] created a baseline for pose estimation through proper pose processing and a simple encoder-decoder neural network.

Since then, graph-based approaches have been gaining popularity, with the emergence of semantic graph convolutional networks (SemGCNs) [18], proposing a way of dealing with the convolution weight sharing problem found in traditional graph convolutional networks, in a way that, after the semantic definition of the graph, each node has its own convolutional matrix.

### Cross-Domain 3D Human Pose Estimation

The idea of using domain adaptation to 3D human pose estimation has been discussed before. Zhang et al. [19], for example, proposed a method in which a synthetic depth-based dataset is used for domain adaptation during the learning step. However, the idea of estimating 3D human pose in a cross-domain scenario was still not discussed by them.

Recent works started to notice the discrepancy in performance between data obtained from distinct distributions [20]. To deal with this issue, several approaches have been proposed, such as that in [21], where synthetic pose datasets artificially generated were used to enhance the amount of data available during the training. Other authors also followed this data augmentation paradigm by using generative adversarial frameworks [22] or a conditional variational auto encoder (CVAE), aiming to generate poses from another dataset distribution [23].

The expansion of the training set through data augmentation was further discussed by recent works aimed at working directly in cross-dataset scenarios, where the discrepancy in performance is even more noticeable. One such work introduced augmentation by adjustment of distinct geometric factors through a joint optimization algorithm trained online [24].

Gholami et al. [25] addressed the domain gap caused by the cross-dataset evaluation through the weakly supervised generation of synthetic 3D motions. These represented the target distribution only looking at the 2D poses, working both as a pose estimation technique and as a synthetic pose generator. A distinct approach that also employs synthetically generated poses, focused on alleviating the domain shift jointly through feature spaces and pose spaces using semantic awareness and skeletal pose adaptation.

The idea of directly using domain adaptation techniques to approach this problem has been discussed in previous works. One such work [26] utilized the skinned multi-person linear (SMPL) model and proposed a method called bilevel online adaptation to reconstruct mesh and pose through a multi-objective optimization problem using temporal constraints to deal with the domain discrepancy.

Chai et al. [27], on the other hand, observed that most of the distribution discrepancy of cross-dataset evaluation stems from camera parameters and the diversity of local structures during training. Thus, they employed domain adaptation by combining a global position alignment mechanism, aiming to eliminate the viewpoint inconsistency, and a local pose augmentation was used to enhance the diversity of the available poses.

The approach proposed by Kundu et al. [28] introduced the usage of uncertainty mechanisms to work with self-supervised 3D human pose estimation. This operated in such a way that minimizing the uncertainty for the unsupervised real dataset alongside a supervised synthetic dataset allows for the cross-dataset pose adaptation. Zhang et al. [29], on the other hand, proposed a method for learning causal representations in order to generate out-of-distribution features that can properly generalize to unseen domains.

Some works tried to solve the problem of pose misrepresentation, which is also found in the literature regarding cross-dataset evaluation. The work Rapczyński et al. [30] aimed to solve this issue through a pose-harmonization mechanism that involved scale normalization and virtual camera augmentation. The approach Sárándi et al. [31], instead, involved using an autoencoder to learn a set of latent keypoints that can properly represent all of the distinct datasets across the same embedding.

Considering the limitations of existing methods in addressing the multifaceted challenges posed by domain discrepancy, often focusing on addressing specific aspects of the domain discrepancy problem, our work aims to introduce a novel approach that tackles this issue from a unified perspective. We propose a domain-unified approach that combines domain adaptation techniques with a universal pose representation and a specialized training technique to mitigate error propagation at the edges.

## 3. Proposed Method

As previously discussed, a few works approached the pose estimation problem in a cross-dataset scenario. However, as exposed in Section 2, this is a multifaceted problem, necessitating the resolution of distinct sub-problems before a valid solution can be considered.

Thus, we have proposed a new method aimed at creating a unified framework for solving the 3D human pose estimation problem in a cross-dataset scenario. To this end, our approach includes three novel modules, each oriented towards addressing specific aspects of the problem, culminating in an integrated framework illustrated in Figure 2. This method is composed of the following components:

**Unified Pose Representation:** To mitigate the challenge of pose misrepresentation found across distinct datasets, we propose an original technique method to find a unified pose representation. This approach involves finding a mapping function Φ that dynamically estimates trajectory vectors between representations;
**Uncertainty Estimation:** Aimed at dealing with the error amplification towards peripheral joints, a phenomenon exacerbated by cross-domain scenarios, we have devised a unique strategy to estimate uncertainty through a naive approach. This involves the usage of an uncertainty loss mechanism by penalizing poses with a high probability of being wrong.
**Domain-Unified Approach Adaptation:** Finally, with the objective of proposing a solution to the domain shift found in cross-dataset scenarios, we incorporate an adversarial-based domain adaptation technique. This mechanism ensures that the feature extraction module finds a uniform representation of the features in a common domain.

Our approach, called Domain Unified Approach (DUA) for 3D Human Pose Estimation, combines the proposed solutions for each sub-problem of cross-dataset 3D human pose estimation into a single framework, in order to increase the robustness of the task.

The following subsections will delve into the specifics of each module, including the motivations behind them, providing a thorough analysis of its implementation and outcomes.

### 3.1. Unified Pose Representation

The incompatibility between pose representations is a commonly observed problem in 3D human pose estimation when dealing with different datasets. Previous works have already discussed this issue in the literature. One such work aims to learn unified representations by utilizing different data sources concurrently [31]. This problem arises from the existence of various body capture sensors and different 3D pose representations being used in the literature causing each dataset to have its own representation.

This problem was previously addressed in the task of volumetric pose shape estimation, by the creation of the Archive of Motion Capture as Surface Shapes (AMASS) [32]. This represents a large and varied database of human motion that unifies 15 different optical-marker-based datasets through the lenses of SMPL, a representation widely used by synthetic datasets in 3D human pose estimation. This is achieved through the usage of the motion and shape capture (MoSh) technique, aimed at estimating body shape SMPL parameters given the 3D pose data [33].

**Figure 3:**
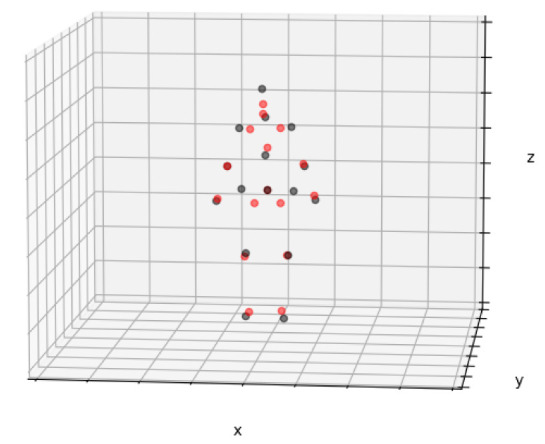
Overlapped joints of the Human3.6M dataset coming from two distinct pose representations, SMPL (red) and the original H3.6M format (black). This makes explicit the difference in the pose representations being used by common 3D human pose datasets in the literature.

An instance elucidating the difference between the moshed SMPL representation of the 3D pose found in the Human3.6M dataset is depicted in Figure 3. In this figure, the SMPL representation (red) has been juxtaposed with the H3.6M representation (black) to accentuate their distinctions, most prominently evident in the regions of the hips and head. The perceptible impact of the pose representation upon the task is undeniable, and unless a common representation is established, the resolution of cross-dataset evaluations remains a challenge.

Therefore, in order to mitigate this problem surrounding pose representations, our approach aims to develop a pose converter used to transform the 3D human pose to the singular pose representation using data obtained from both the SMPL and original Human3.6M representations.

This issue has already been discussed in the literature [30]. However, the authors tried to find a harmonization and normalization technique through handcrafted features, which does not preserve the body proportions after normalization. Thus, we proposed to create a pose converter to dynamically learn how to convert from one pose representation to another.

The idea of the proposed converter network is to dynamically find an array, based on the network weights and the 3D pose input, that is added to the pose results in the corresponding SMPL format.

The proposed converter network operates by dynamically identifying an array based on the 3D pose input. In mathematical terms, the mapping function Φ:A↦B takes a set of joints $X_{A}$ represented in pose format A and calculates weights to map $X_{A}$ to a representation $X_{B}$ in the B pose format.

Instead of directly mapping A to B, the task of converting between representation spaces of the same semantic skeleton graph involves finding trajectory vectors that describe the new joint positions and their trajectories in the new pose space. To simplify this process, we work directly with the joint trajectory vectors by introducing a mapping function φ:A↦(B−A), in such a way that:

(1) $$X_{B}=\varphi \left(X_{A}\right)+X_{A}.$$

**Figure 4:**
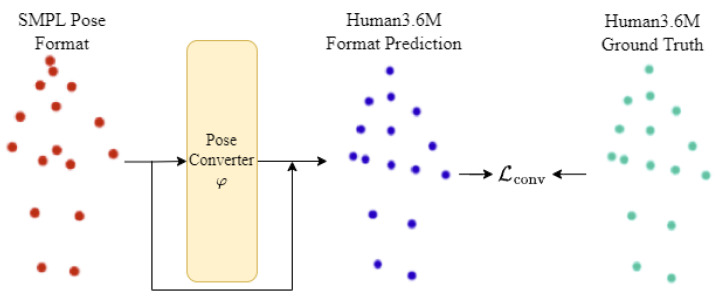
Pose conversion method used to find a unified pose representation.

The weights of the mapping function φ are obtained through a single-layer residual neural network using gradient descent. To train this network, a loss function combining mean squared error and mean average error is employed. The loss is given by:

(2) $$L_{conv}=\alpha {\left(X_{B}-X_{A}\right)}^{2}+\left(1-\alpha \right)∥X_{B}-X_{A}∥,$$

where 0≤α≤1 is a hyperparameter used to impose the importance of each loss term. Figure 4 illustrates the learning process of the proposed method.

### 3.2. Pose Uncertainty

Three-dimensional (3D) human pose estimation presents a challenge in the form of error propagation within the most extreme kinematic group, compounded by the ill-defined monocular estimation resulting from self-occlusion during varying camera perspectives. In order to mitigate this problem, an approach has been devised to quantify and reduce the uncertainties arising from such scenarios.

Uncertainty in Bayesian networks was defined in two forms: epistemic uncertainty captures the model’s ignorance despite sufficient training data with well-defined data distributions, while aleatory uncertainty aims to model unexplained uncertainties within the current training data [34]. Previous works have explored uncertainty modeling through Bayesian networks for 3D human pose estimation using different approaches [28,35]. In this work, we propose a method based on a naïve definition of uncertainty.

To quantify uncertainty, our method utilizes the features extracted from the pose estimator to predict the probability of a joint being incorrect. A random variable U is generated by mapping the normalized Euclidean distance of the joint difference, where joints with small distances are mapped near zero and those with significant distances are mapped to one. This mapping allows for improved assessment and quantification of uncertainty associated with individual joints. The Uncertainty Module on Figure 2 illustrates our devised approach using the method proposed by Martinez et al. [9] as the backbone.

Our method consists of *J* heads, each representing one joint in the pose representation. After passing through the sigmoid activation function, each head provides the probability of a specific joint being incorrect. This probability is learned through supervised training by comparing the output to the normalized Euclidean distance. The Uncertainty Error is calculated as the L1 distance between the array composed of the heads and the normalized distance.

Given the outputs of a pose feature extractor θ for an input *x*, inserted into each of the *J* heads $H_{j}$, and using a pose estimator head P to obtain the output 3D pose, we define U(x) as the desired pose uncertainty obtained by concatenating each head $H_{j}$. In other words, using the concatenation operation denoted by || and the sigmoid function σ, we have:

(3) $$U\left(x\right)=||\sigma (H_{j}\left(\theta \left(x\right)\right)).$$

By combining the pose feature extractor and the pose estimator head through the function Π(x)=P(θ(x)), where *P* represents the pose estimator, the uncertainty U(x) of a given pose *x* can be learned. This is achieved by comparing the normalized Euclidean distance of each joint in the predicted pose Π(x) to the ground-truth 3D pose *y*, and comparing it to the output uncertainty using L1 distance. The loss function for training is defined as:

(4) $$L_{unc}\left(x\right)=∥\frac{{\left(\sqrt{\Pi (x)-y}\right)}^{2}}{{∥\Pi \left(x\right)∥}_{2}{∥y∥}_{2}}-U\left(x\right)∥.$$

### 3.3. Domain Adaptation

When data spanning from the training dataset and the test dataset show differences between their data distributions, a problem called domain shift arises. This can cause a negative impact on the accuracy of classifiers, causing images to be misclassified [36]. One way to deal with this problem is to use domain adaptation techniques [37].

Domain adaptation refers to a sub-area of transfer learning, whose goal is to use data from a domain other than the one used for training, in order to improve the accuracy of the classifier when applied to an alternative dataset [15]. The formal definition of the concepts that compose the basis of domain adaptation theory, according to Pan and Yang [38] are exposed hereafter.

**Definition** **1** (Domain)**.** *A domain D is composed of a feature space F with d dimensions and a marginal probability function P(x), which means that D={F,P(x)}, with x∈F.*

**Definition** **2** (Task)**.** *A domain D, a task T consists of a set of labels Y and a classifier f(x), which means that T={Y,f(x)}, with y∈Y and f(x)=P(y|x).*

**Definition** **3** (Domain Adaptation)**.** *Given a source domain $D_{S}$ and a target domain $D_{T}$, and assuming that $D_{S}\neq D_{T}$ regarding their marginal probabilities $P\left(X^{S}\right)\neq P\left(X^{T}\right)$, and two tasks $T_{S}\approx T_{T}$, with conditional distribution $P\left(Y^{S}|X^{S}\right)\approx P\left(Y^{T}|X^{T}\right)$, the goal of domain adaptation is to improve the prediction $f_{T}\left(\cdot \right)$ in the target domain $D_{T}$ using the source domain $D_{S}$ data.*

### 3.4. Domain-Unified Approach (DUA)

Recent advancements in deep learning enabled the development of architectures and training protocols specifically focused on domain adaptation in the deep learning setting [15]. Initially, deep neural networks were used solely as feature extractors, followed by the application of traditional domain adaptation techniques. However, advancements in the field led to architectures that directly address domain adaptation challenges.

These architectures take various forms, including methods based on domain discrepancy, adversarial training, autoencoders, and spatial relationships within the data. Considering the context of pose estimation, our objective is to tackle the following problem:

**Problem** **1** (Domain-Adaptive 3D Human Pose Estimation)**.** *Given a source domain $D_{S}$ composed of a set of poses X,Y and an unsupervised target domain $D_{T}$ consisting of a set of pose annotations X, with distinct marginal probabilities $D_{S}\neq D_{T}$, the goal is to find a feature map θ and a pose estimator head P such that the conditional probability $P\left(\theta \left(X_{S}\right)|X_{S}\right)\approx P\left(\theta \left(X_{T}\right)|X_{T}\right)$ without negatively affecting the efficacy of the pose regressor head P.*

In this context, our pose estimator should be capable of accurately inferring poses from both domains with minimal error. In order to address the Problem 1, we proposed the Domain-Unified Approach (DUA), a method capable of accurately inferring poses from source and target domains with minimal error, by combining a pose conversion unit, presented in Section 3.1, and the uncertainty loss mechanism, described in Section 3.2, with a domain adaptation module. The general idea of DUA is to maximize the distance between 3D human poses using a domain discriminator that is jointly optimized with the entire deep learning system.

To achieve this, we employ an architecture inspired by domain adversarial neural networks (DANNs) [39]. To find the desired pose, given a pose estimator Π, the following pose loss is used:

(5) $$L_{pose}\left(x\right)=\beta {\left(y-\Pi \left(x\right)\right)}^{2}+\left(1-\beta \right)∥y-\Pi \left(x\right)∥,$$

where 0≤β≤1 is a hyperparameter that controls the importance of each part of the pose loss.

The pose estimator is engaged in a minimax game, aiming to minimize $L_{\mathrm{pose}}$ while simultaneously maximizing the domain discrepancy of the joints to find the optimal representation from the pose feature extractor θ. This is achieved using a domain classifier *G*, trained with the loss:

(6) $$L_{d}\left(x\right)=G\left(\theta \left(x\right)\right)\log\left(G\left(\theta \left(x\right)\right)\right)+\left(1-G\left(\theta \left(x\right)\right)\right)log\left(1-G\left(\theta \left(x\right)\right)\right).$$

In our approach, the unified pose representation obtained by the converter is pre-trained, and its weights remain frozen during training. On the other hand, the other components of the method are trained in an online mode. The overall training loss is given by:

(7) $$L=\lambda L_{d}+\gamma L_{unc}+L_{pose},$$

where 0<λ<1 and 0<γ<1 are regularization parameters.

## 4. Experimental Setting

This section aims to present the training and evaluation protocol, as well as the experimental environment in which our method was assessed. Furthermore, this section highlights the specific metrics and datasets utilized to conduct the experiments.

All our experiments were conducted using a computer with two Intel Xeon E5620 CPUs (Santa Clara, CA, USA), 48 GB of RAM, and an NVIDIA TitanXP GPU with 12 GB of VRAM (Santa Clara, CA, USA). During training, a batch size of 2048 was employed, with a learning rate of $1\times 10^{-3}$ paired with the Adam optimizer. For hyperparameters, α=0.5 were employed in the pose conversion scenario, for the pose estimator, λ=0.01, γ=0.1, and β=0.4 were chosen via empiric evaluation. The pose conversion was pre-trained and its weights were frozen on the DUA method. Further details on each module can be found in Section 5.

### 4.1. Evaluation Protocol

Cross-dataset evaluation in 3D human pose estimation shows a significant challenge due to the inherent misalignment of target distributions, especially when synthetic data are involved. The scarcity of literature addressing this specific scenario has motivated only a few authors to explore the evaluation protocol for assessing cross-domain generalization when synthetic data are involved [28,29].

In this work, our focus lies on evaluating the performance of synthetic to real cross-domain pose estimation. Building upon previous works, we adopt a widely used general domain adaptation evaluation protocol to assess the effectiveness of domain generalization. Specifically, we employ an unsupervised training approach using the target dataset training split, while utilizing the supervised source data for training. The evaluation is conducted using both synthetic and real datasets as source data.

For the purpose of comparison, we adopt the unified pose representation of the Human3.6M model as our baseline, with the pose converter trained to transform the SMPL pose representation of the dataset into the Human3.6M representation. By employing this unified pose representation, we aim to facilitate meaningful comparisons with existing approaches.

In the following sections, we will provide a description of the datasets and metrics utilized in our experimental evaluation.

### 4.2. Datasets

**Figure 5:**
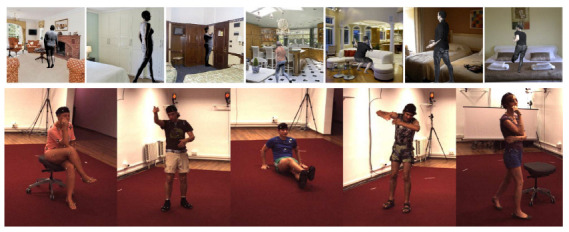
Example images found in the SURREAL (**first row**) and Human3.6M (**second row**) datasets.

We employed two datasets in our cross-domain experiments: SURREAL [40] and Human3.6M [41]. In particular, the SURREAL dataset was used to represent the synthetic image domain, while the Human3.6M dataset was used to represent the real people image domain. Figure 5 shows examples of images found in both datasets.

**SURREAL:** It is a large-scale dataset containing more than 6 million photorealistic synthetic image frames found in real environments with large variations in texture, body time, camera positioning, and pose actions. The dataset contains information about the depth map, body parts, optical flow, and 2D and 3D joints;
**Human3.6M:** The Human3.6M dataset is composed of real people images obtained from a Motion Capture system based on markers. It contains scenes of 11 professional actors obtained in a controlled environment. The dataset has about 3.6 million annotations of 3D poses, considering four different angles. This dataset also has three evaluation protocols with different data for training and testing.

### 4.3. Metrics

The methods proposed and developed in this work were evaluated by the standard metrics applied in the literature in the 3D human pose estimation problem. Among those metrics, we mention:

**MPJPE:** The mean per-joint position error (MPJPE) represents the mean error, in millimeters, between the estimated points and the real points after the root joint alignment;
**P-MPJPE:** The Procrustes-aligned mean per-joint position error (P-MPJPE) represents the mean error, in millimeters, between the estimated points and the real points after Procrustes alignment.

## 5. Results

In this section, we present the results of our 3D human pose estimation method, which was evaluated using the protocol discussed in Section 4. Our method consists of three essential modules: pose conversion, pose uncertainty, and a domain-unified approach. To provide a comprehensive analysis, we showcase the results of each module in their respective subsections. By evaluating the performance of each module individually, we gain insights into their effectiveness and contribution toward accurate and robust pose estimation.

### 5.1. Unified Pose Representation

Our Unified Pose Representation technique described in Section 3.1 was trained for 100 epochs, and the converted poses were evaluated using the MPJPE (Protocol 1) and P-MPJPE (Protocol 2) metrics on the Human3.6M dataset. We conducted evaluations by joint and by action. The results, shown in Table 1 and Table 2, clearly demonstrate the significant error reduction achieved by the conversion method when compared to SMPL poses without conversion. This error reduction has a particular impact when predicting poses from different domains.

The results presented in Table 1 demonstrate a notable reduction in the mean per-joint position error (MPJPE) across all action groups when comparing the converted poses with their original counterparts. This significant decrease highlights the detrimental effects of pose misrepresentation and the positive impact of utilizing a properly converted pose through our method. By effectively converting the poses to a more appropriate representation, our method successfully mitigates the negative effects of misrepresentation, leading to improved pose estimation performance.

Furthermore, the results presented in Table 2 demonstrate the effectiveness of our method in achieving a proper pose representation across all individual joints. The observed reduction in error for each joint indicates the successful correction of mispositioning issues and highlights the ability of our approach to mitigate error propagation from previous representations. The largest errors per joint, previously contained within misrepresented poses are now dislocated to edge joints, which aligns with the expected behavior in normal pose estimation.

**Figure 6:**
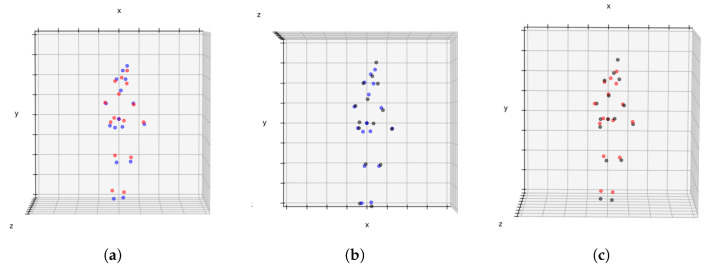
Overlapping of the different pose representations available, Human3.6M (red), SMPL ground-truth (blue), converted pose (black). Item (**a**) shows a Human3.6M (red dots) pose superimposed on the original SMPL pose (blue dots). Item (**b**) shows the resulting pose after conversion (black dots) superimposed to the original SMPL pose (blue dots). Item (**c**) shows the converted pose (black dots) superimposed to the original Human3.6m pose (red dots).

In Figure 6, we provide visual comparisons to illustrate the effectiveness of our pose conversion method. On the left, a Human3.6M pose (depicted by red dots) is superimposed on the SMPL pose without conversion (depicted by blue dots). In the middle, the resulting conversion (depicted by black dots) is superimposed on the original SMPL pose (blue dots). On the right, we compare the Human3.6M pose (red dots) to the SMPL pose converted to the Human3.6M format using our method (black dots). It can be noted that the conversion of the SMPL pose to the Human3.6M pose model helps to reduce errors, particularly in the hip area, thus improving the overall pose estimation accuracy and alignment with the target pose format.

It is evident from the visual comparisons that the pose conversion successfully aligns the SMPL poses with the target format, effectively capturing the key joint positions and maintaining the overall pose structure. The converted poses exhibit improved similarity to the ground-truth poses, indicating the accuracy and fidelity of the conversion process. This qualitative analysis further corroborates the quantitative results, demonstrating the efficacy of the pose conversion method in achieving a more accurate and compatible representation for 3D human pose estimation tasks.

### 5.2. Pose Uncertainty

Our proposed approach was trained on 200 epochs for each representation. The results obtained are shown in Table 3. Results were evaluated in two scenarios, using a linear backbone and a graph-based backbone, and with three types of 2D input information: ground truth, stacked hourglass, and a more robust 2D pose extractor based on cascaded pyramid networks.

The experimental results demonstrate that our approach surpasses the performance of their respective backbones, providing better results in almost all the action groups, and effectively mitigating the performance gap between graph-based and linear methods. However, it is important to note that our method is still dependent on the choice of a backbone architecture, as it inherits and improves upon the errors already present in certain action groups from the backbone. In some cases, the original backbones still outperformed our method, but on average, our approach resulted in lower errors. Moreover, we observed that selecting a more accurate 2D human pose estimator significantly enhanced the performance of our method, indicating the importance of leveraging reliable 2D pose information in the overall pose estimation process.

Overall, our results validate the effectiveness of incorporating pose uncertainty estimation into the 3D human pose estimation pipeline, leading to enhanced accuracy and robustness in capturing human poses across different action categories.

### 5.3. Domain-Unified Approach

Our proposed technique aims to unify our previously proposed methods by combining the pre-trained pose converter (with frozen weights), the pose uncertainty module, and the domain adaptation protocol. The domain adaptation method was trained online for 300 epochs, and an evaluation was conducted using both the SURREAL and Human3.6M datasets as source data. Results of the domain adaptation method are presented in Table 4.

Notably, the utilization of domain adaptation significantly mitigates the problem caused by domain discrepancy, when evaluating the most practical scenario, of training with a huge synthetic dataset and applying it to a real-world scenario (SURREAL → Human3.6M), our method leads to a reduction of 44.1 mm in the mean per-joint position error (MPJPE). This improvement surpasses the current state-of-the-art in the task. Additionally, we included the SURREAL data without conversion only in the domain adaptation scenario to compare and evaluate the effectiveness of the converted pose in the overall method results.

The impact of employing pose conversions is evident in the Human3.6M → SURREAL scenario, where the evaluation is conducted on a larger dataset comprising a distinct subset of actions. In this challenging scenario, the performance of our method with domain adaptation experiences a slight decrease with domain adaptation. This can be attributed to the misrepresentation of poses, which results in difficulty in the optimization of our domain discriminator. However, by incorporating pose conversion into our method, we are able to mitigate this discrepancy and observe a performance improvement effectively.

Furthermore, when compared to other state-of-the-art methods employed in the real-to-synthetic cross-dataset scenario (as shown in Table 5), our method outperforms all previously proposed techniques. One significant advantage of our approach is the use of a universal pose representation, wherein the conversion step mitigates issues arising from variations in body capture sensors across different datasets.

Ablation results are made explicit in Table 6. This table effectively illustrates the importance of each component of our method. Specifically, the unified pose representation emerges as the primary contributor to addressing the problem. Conversely, adopting domain adaptation in isolation, without a common representation causes a negative impact on the results. Interestingly, no such adverse influence is observed when solely employing the shared pose representation. However, this negative impact is not seen when both methods are combined, with a clear enhancement in results becoming evident.

**Figure 7:**
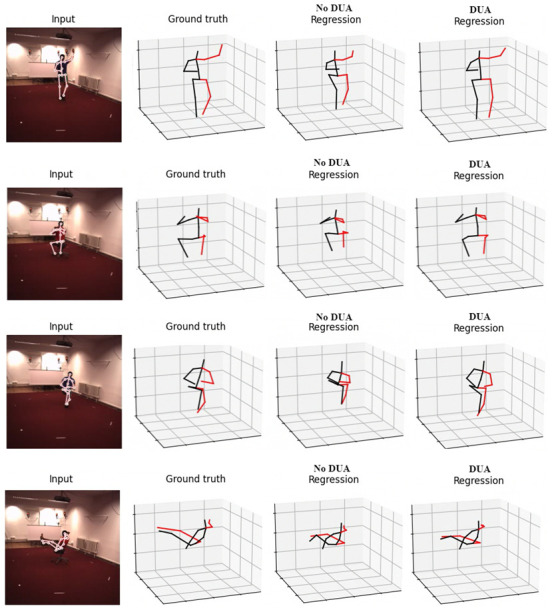
Qualitative comparison of the results obtained by our method (DUA) in the Human3.6M dataset, in comparison to a cross-dataset scenario without the proposed domain adaptation technique.

We also observed that the pose conversion step substantially impacts our method’s overall performance. This finding highlights the importance of finding a unified pose representation to mitigate domain discrepancy. Qualitative results are presented in Figure 7, which clearly demonstrates the consequences of not applying domain adaptation, resulting in significant distortions in certain pose regions, including body proportion loss and mispositioning of joints, such as the hips.

It is worth noting that the performance of the same-domain scenario exhibits a slight decrease in efficacy when employing domain adaptation techniques. This can be attributed to the objective of our approach, which aims to obtain a more generalized set of pose features through domain adaptation. By doing so, our method mitigates the risk of overfitting to specific actions or camera angles, as previously discussed in the literature [14].

Although there is a slight decrease in performance in the same-domain scenario, the overall benefit of achieving improved generalization and robustness across different domains outweighs this slight trade-off.

## 6. Conclusions

In conclusion, our proposed technique successfully addressed key challenges in 3D human pose estimation by integrating the pre-trained pose converter, the pose uncertainty module, and the domain adaptation protocol. Through the application of domain adaptation, we have effectively tackled the issue of domain discrepancy, leading to a remarkable reduction of 44.1mm (29.24%) in mean per-joint position error MPJPE, when training with the synthetic dataset SURREAL and evaluating with Human3.6M. This substantial improvement surpasses the current state-of-the-art in the task, highlighting the efficacy of our domain adaptation method.

By utilizing a universal pose representation and incorporating the pose conversion step, we effectively addressed challenges arising from variations in body capture sensors across different datasets. This capability enhances the adaptability and generalization of our approach, providing robust and accurate pose estimation results.

Our method corroborates with results from previous works [28,29], which assert the existence of a problem regarding out-of-distribution unseen data and the possibility of using domain adaptation as a way to increase robustness in these scenarios. Furthermore, our method expands on existing techniques by not only tackling the challenge of domain adaptation but also by addressing the intricacies of pose misrepresentation. By establishing a Unified Pose Representation for cross-domain data and effectively integrating domain adaptation, we substantively enhance the outcomes, thereby contributing to the advancement of the field.

Although there is still room for further advancements in the field of 3D human pose estimation, such as improving the backbone estimation or enhancing the quality of uncertainty estimation, our method represents a significant step forward in improving the accuracy and robustness of pose estimation by the combination of our pose conversion, pose uncertainty estimation, and domain adaptation modules.

## Figures and Tables

**Table 1:** Comparison of the MPJPE and P-MPJPE performance error by action (mm—the lower the better) of the SMPL model without conversion (right) in comparison to the converted pose obtained with our method (left), when overlapped to the original Human3.6M pose.

Action	MPJPE SMPL	P-MPJPE SMPL	MPJPE Conversion	P-MPJPE Conversion
Direct.	63.84	60.66	28.95	21.81
Discuss	69.74	60.04	35.19	26.59
Eating	70.95	60.04	33.72	26.07
Greet	63.18	59.99	28.90	23.13
Phone	71.20	61.81	36.77	29.83
Photo	68.89	62.11	32.77	26.06
Pose	63.29	60.25	29.68	23.77
Purch.	72.37	60.47	34.97	24.33
Sitting	77.79	62.63	38.93	31.20
SittingD.	81.35	64.39	42.91	32.94
Smoke	71.38	62.59	34.29	27.42
Wait	66.40	60.06	33.23	26.12
WalkD.	69.82	60.01	35.47	26.16
Walk	62.24	59.10	30.51	24.24
WalkT.	61.74	59.15	29.05	23.86
Avg.	68.95	60.89	33.70	26.24

**Table 2:** Per-joint error (mm—the lower the better) when comparing the converted pose using our method (left side) and the unconvertedSMPL poses (right side) with the original Human3.6M pose.

Joint	MPJPE SMPL	P-MPJPE SMPL	MPJPE Conversion	P-MPJPE Conversion
Hip	00.00	20.89	00.00	18.09
Right Hip	125.03	122.87	14.81	26.84
Right Knee	44.73	44.61	34.72	29.37
Right Foot	47.09	29.11	41.28	28.71
Left Hip	104.66	104.26	18.62	19.88
Left Knee	49.04	47.62	39.96	34.10
Left Foot	63.97	46.92	49.60	31.48
Spine	67.05	89.22	28.48	16.83
Thorax	71.94	62.84	35.18	24.20
Head	118.46	99.80	54.31	44.69
Left Shoulder	106.59	88.95	39.91	31.98
Left Elbow	60.27	35.68	41.93	23.66
Left Wrist	44.09	28.21	27.07	15.80
Right Shoulder	105.49	90.97	43.43	31.86
Right Elbow	55.11	37.31	42.07	26.76
Right Wrist	39.19	28.52	29.89	17.91

**Table 3:** Results of the MPJPE error (mm—the lower the better) obtained from the 3D human pose considering all of the established scenarios with distinct 2D pose sources: stacked hourglass (HG) (denoted by *), cascaded pyramid networks (CPNs) (denoted by †), and ground truth (GT) (denoted by ‡). Experiments were performed using a linear backbone (indicated by ★) and a graph-based backbone (indicated by *§*). The best results are presented in bold.

	[9] ${}^{*}$	[18] ${}^{*}$	Ours ${}^{*}$	Ours ${}^{*}$	Ours ${}^{†★}$	Ours ${}^{†§}$	[18] ${}^{‡}$	Ours ${}^{‡★}$	Ours ${}^{‡§}$
Direct.	51.8	48.2	50.9	50.1	**46.2**	46.4	37.8	**34.8**	41.0
Discuss	56.2	60.8	54.5	56.1	**50.4**	52.1	49.4	**42.4**	46.2
Eating	58.1	51.8	56.0	58.0	**51.1**	51.8	37.6	35.6	**34.4**
Greet	59.0	64.0	57.0	56.7	52.7	**52.2**	40.9	**39.9**	**39.9**
Phone	69.5	64.6	65.7	63.5	57.3	**56.5**	45.1	42.2	**38.1**
Photo	78.4	**53.6**	72.6	73.1	67.8	67.0	**41.4**	50.3	46.6
Pose	55.2	51.1	52.6	52.4	**50.5**	51.5	**40.1**	42.6	44.3
Purch.	58.1	67.4	54.5	54.4	**48.2**	49.0	48.3	**37.0**	39.1
Sitting	74.0	88.7	70.2	71.1	**64.1**	64.7	50.1	49.5	**46.0**
SittingD.	94.6	**57.7**	89.4	93.5	72.6	73.7	**42.2**	54.0	52.4
Smoke	62.3	73.2	59.7	60.2	53.7	**53.3**	53.5	**40.3**	39.5
Wait	59.1	65.6	57.4	57.8	**51.3**	51.4	44.3	**41.6**	43.5
WalkD.	65.1	48.9	62.9	61.7	57.2	**55.1**	40.5	42.6	**40.4**
Walk	49.5	64.8	47.3	47.8	41.8	**41.5**	47.3	32.1	**31.5**
WalkT.	52.4	51.9	51.5	51.9	46.7	**45.2**	39.0	35.0	**34.2**
Avg.	62.9	60.8	60.2	60.6	**54.1**	**54.1**	43.8	41.3	**41.1**

**Table 4:** Results from the MPJPE metric (mm—the lower the better) obtained from different domain adaptation scenarios.

	Target Dataset					
**Source Dataset**	**SURREAL**		**Converted SURREAL**		**Human3.6M**	
	**MPJPE**	**P-MPJPE**	**MPJPE**	**P-MPJPE**	**MPJPE**	**P-MPJPE**
Human3.6M (No DA)	107.6 mm	65.7 mm	100.3 mm	62.6 mm	41.3 mm	32.7 mm
SURREAL (No DA)	-	-	40.4 mm	29.0 mm	150.8 mm	88.2 mm
Human3.6M + DUA	108.9 mm	70.1 mm	96.1 mm	57.9 mm	74.0 mm	52.2 mm
SURREAL + DUA	-	-	55.8 mm	37.7 mm	106.7 mm	65.6 mm

**Table 5:** Quantitative results obtained on the H3.6M→SURREAL evaluation. Table results and layout are obtained from experiments conducted by [28,29], and bold indicates the best result.

Method	H3.6M→SURREAL	
	MPJPE	P-MPJPE
DDC [42]	117.5	80.1
DAN [43]	114.2	78.4
DANN [39]	113.6	77.2
Zhang et al. [29]	103.3	69.1
Kundu et al. [28]	99.6	67.2
Kundu et al. [28] *	96.4	65.1
DUA (Ours)	**96.1**	**57.9**

* Denotes an alternative approach for cross-domain evaluation conducted using test-time adaptation.

**Table 6:** Explicit ablation results of our method evaluated on the Human3.6M→SURREAL scenario.

Method	MPJPE
w/o Unified Pose Representation and DA	107.6 mm
w/o Unified Pose Representation	108.9 mm
w/o DA	100.3 mm
DUA	96.1 mm

## Data Availability

We will provide links to the datasets used in our experiments. Human3.6M: http://vision.imar.ro/human3.6m/description.php (accessed on 18 June 2023). SURREAL: https://www.di.ens.fr/willow/research/surreal/data/ (accessed on 18 June 2023). Further data can be made available on request.

## Abbreviations

The following abbreviations are used in this manuscript:

AMASS	Archive of Motion Capture As Surface Shapes
CPN	Cascaded Pyramid Network
CVAE	Conditional Variational Auto Encoder
DA	Domain Adaptation
DAN	Deep Adaptation Network
DANN	Domain Adversarial Neural Network
DDC	Deep Domain Confusion
DUA	Domain Unified Approach
GT	Ground Truth
H3.6M	Human3.6M
HG	Stacked Hourglass
MoSh	Motion and Shape capture
MPJPE	Mean Per-Joint Position Error
P-MPJPE	Procrustes-Aligned Mean Per-Joint Position Error
SemGCN	Semantic Graph Convolutional Network
SMPL	Skinned Multi-Person Linear Model

## References

[1] Zheng C., Wu W., Yang T., Zhu S., Chen C., Liu R., Shen J., Kehtarnavaz N., Shah M. (2020). Deep Learning-Based Human Pose Estimation: A Survey. arXiv.

[2] Chen Y., Tian Y., He M. (2020). Monocular Human Pose Estimation: A Survey of Deep Learning-Based Methods. Comput. Vis. Image Underst..

[3] Doersch C., Zisserman A. (2019). Sim2real Transfer Learning for 3D Human Pose Estimation: Motion to the Rescue. arXiv.

[4] Bartol K., Bojanic D., Petkovic T., D’Apuzzo N., Pribanic T. A Review of 3D Human Pose Estimation from 2D Images. Proceedings of the 3DBODY.TECH 2020—11th International Conference and Exhibition on 3D Body Scanning and Processing Technologies.

[5] Lin T.Y., Maire M., Belongie S., Hays J., Perona P., Ramanan D., Dollár P., Zitnick C.L., Fleet D., Pajdla T., Schiele B., Tuytelaars T. (2014). Microsoft COCO: Common Objects in Context. Computer Vision—ECCV 2014.

[6] Cao Z., Hidalgo G., Simon T., Wei S.E., Sheikh Y. (2019). OpenPose: Realtime multi-person 2D pose estimation using Part Affinity Fields. IEEE Trans. Pattern Anal. Mach. Intell..

[7] Kreiss S., Bertoni L., Alahi A. Pifpaf: Composite fields for human pose estimation. Proceedings of the IEEE/CVF Conference on Computer Vision and Pattern Recognition.

[8] Newell A., Yang K., Deng J., Leibe B., Matas J., Sebe N., Welling M. (2016). Stacked Hourglass Networks for Human Pose Estimation. Computer Vision—ECCV 2016.

[9] Martinez J., Hossain R., Romero J., Little J.J. A Simple Yet Effective Baseline for 3d Human Pose Estimation. Proceedings of the 2017 IEEE International Conference on Computer Vision (ICCV).

[10] Wei G., Lan C., Zeng W., Chen Z. (2019). View invariant 3d human pose estimation. IEEE Trans. Circuits Syst. Video Technol..

[11] Véges M., Varga V., Lorincz A. (2019). 3D human pose estimation with siamese equivariant embedding. Neurocomputing.

[12] Xu J., Yu Z., Ni B., Yang J., Yang X., Zhang W. Deep kinematics analysis for monocular 3d human pose estimation. Proceedings of the IEEE/CVF Conference on Computer Vision and Pattern Recognition.

[13] Xu Y., Wang W., Liu T., Liu X., Xie J., Zhu S.C. (2021). Monocular 3D Pose Estimation via Pose Grammar and Data Augmentation. IEEE Trans. Pattern Anal. Mach. Intell..

[14] Chen T., Fang C., Shen X., Zhu Y., Chen Z., Luo J. (2021). Anatomy-aware 3D Human Pose Estimation with Bone-based Pose Decomposition. IEEE Trans. Circuits Syst. Video Technol..

[15] Csurka G. (2017). Domain Adaptation in Computer Vision Applications.

[16] Stamou G., Krinidis M., Loutas E., Nikolaidis N., Pitas I. (2005). 2D and 3D Motion Tracking in Digital Video. Handbook of Image and Video Processing.

[17] Moreno-Noguer F. 3d human pose estimation from a single image via distance matrix regression. Proceedings of the IEEE Conference on Computer Vision and Pattern Recognition.

[18] Zhao L., Peng X., Tian Y., Kapadia M., Metaxas D.N. Semantic graph convolutional networks for 3d human pose regression. Proceedings of the IEEE/CVF Conference on Computer Vision and Pattern Recognition.

[19] Zhang X., Wong Y., Kankanhalli M.S., Geng W. Unsupervised domain adaptation for 3D human pose estimation. Proceedings of the 27th ACM International Conference on Multimedia.

[20] Manesco J.R.R., Marana A.N. A Survey of Recent Advances on Two-Step 3D Human Pose Estimation. Proceedings of the Brazilian Conference on Intelligent Systems.

[21] Dabral R., Mundhada A., Kusupati U., Afaque S., Sharma A., Jain A. Learning 3d human pose from structure and motion. Proceedings of the European Conference on Computer Vision (ECCV).

[22] Yang W., Ouyang W., Wang X., Ren J., Li H., Wang X. 3d human pose estimation in the wild by adversarial learning. Proceedings of the IEEE Conference on Computer Vision and Pattern Recognition, Salt Lake City.

[23] Jiang Y., Liu X., Wu D., Zhao P. (2021). Residual Deep Monocular 3D Human Pose Estimation using CVAE synthetic data. J. Phys. Conf. Ser..

[24] Gong K., Zhang J., Feng J. Poseaug: A differentiable pose augmentation framework for 3d human pose estimation. Proceedings of the IEEE/CVF Conference on Computer Vision and Pattern Recognition.

[25] Gholami M., Wandt B., Rhodin H., Ward R., Wang Z.J. Adaptpose: Cross-dataset adaptation for 3d human pose estimation by learnable motion generation. Proceedings of the IEEE/CVF Conference on Computer Vision and Pattern Recognition.

[26] Guan S., Xu J., Wang Y., Ni B., Yang X. Bilevel online adaptation for out-of-domain human mesh reconstruction. Proceedings of the IEEE/CVF Conference on Computer Vision and Pattern Recognition.

[27] Chai W., Jiang Z., Hwang J.N., Wang G. (2023). Global Adaptation meets Local Generalization: Unsupervised Domain Adaptation for 3D Human Pose Estimation. arXiv.

[28] Kundu J.N., Seth S., YM P., Jampani V., Chakraborty A., Babu R.V. Uncertainty-aware adaptation for self-supervised 3d human pose estimation. Proceedings of the IEEE/CVF Conference on Computer Vision and Pattern Recognition.

[29] Zhang X., Wong Y., Wu X., Lu J., Kankanhalli M., Li X., Geng W. Learning causal representation for training cross-domain pose estimator via generative interventions. Proceedings of the IEEE/CVF International Conference on Computer Vision.

[30] Rapczyński M., Werner P., Handrich S., Al-Hamadi A. (2021). A baseline for cross-database 3d human pose estimation. Sensors.

[31] Sárándi I., Hermans A., Leibe B. Learning 3D Human Pose Estimation from Dozens of Datasets using a Geometry-Aware Autoencoder to Bridge Between Skeleton Formats. Proceedings of the IEEE/CVF Winter Conference on Applications of Computer Vision.

[32] Mahmood N., Ghorbani N., Troje N.F., Pons-Moll G., Black M.J. AMASS: Archive of Motion Capture as Surface Shapes. Proceedings of the International Conference on Computer Vision.

[33] Loper M.M., Mahmood N., Black M.J. (2014). MoSh: Motion and Shape Capture from Sparse Markers. Acm Trans. Graph..

[34] Kendall A., Gal Y., Cipolla R. Multi-task learning using uncertainty to weigh losses for scene geometry and semantics. Proceedings of the IEEE Conference on Computer Vision and Pattern Recognition, Salt Lake City.

[35] Li H., Shi B., Dai W., Zheng H., Wang B., Sun Y., Guo M., Li C., Zou J., Xiong H. (2023). Pose-Oriented Transformer with Uncertainty-Guided Refinement for 2D-to-3D Human Pose Estimation. arXiv.

[36] Kouw W.M., Loog M. (2018). An introduction to domain adaptation and transfer learning. arXiv.

[37] Patel V.M., Gopalan R., Li R., Chellappa R. (2015). Visual domain adaptation: A survey of recent advances. IEEE Signal Process. Mag..

[38] Pan S.J., Yang Q. (2010). A Survey on Transfer Learning. IEEE Trans. Knowl. Data Eng..

[39] Ganin Y., Ustinova E., Ajakan H., Germain P., Larochelle H., Laviolette F., Marchand M., Lempitsky V. (2016). Domain-Adversarial Training of Neural Networks. J. Mach. Learn. Res..

[40] Varol G., Romero J., Martin X., Mahmood N., Black M.J., Laptev I., Schmid C. Learning from Synthetic Humans. Proceedings of the CVPR.

[41] von Marcard T., Henschel R., Black M.J., Rosenhahn B., Pons-Moll G., Ferrari V., Hebert M., Sminchisescu C., Weiss Y. (2018). Recovering Accurate 3D Human Pose in the Wild Using IMUs and a Moving Camera. Computer Vision—ECCV 2018.

[42] Tzeng E., Hoffman J., Zhang N., Saenko K., Darrell T. (2014). Deep domain confusion: Maximizing for domain invariance. arXiv.

[43] Long M., Cao Y., Wang J., Jordan M.I. Learning Transferable Features with Deep Adaptation Networks. Proceedings of the ICML’15: 32nd International Conference on International Conference on Machine Learning.

